# THE LONG-TERM EFFECTS OF AIR POLLUTION ON ELDERLY HEALTH: AN EMPIRICAL STUDY FROM CHINA

**DOI:** 10.1093/geroni/igae098.0760

**Published:** 2024-12-31

**Authors:** Xiaotong Tang, Hong Mi

**Affiliations:** Zhejiang University, Hangzhou, Zhejiang, China (People’s Republic); Zhejiang University, Hangzhou, Zhejiang, China (People’s Republic)

## Abstract

Healthy elderly individuals are assets to families, communities, and the economic development. Identifying and quantifying long-term effects of environmental factors on elderly health could bring new evidence and policy insights for optimizing pollution response, promoting healthy aging, and unlocking a second demographic dividend. This paper matches city air pollution data from 2003 to 2020 with individual data taken from the China Health and Retirement Longitudinal Study (CHARLS) for the years 2013 to 2020. By utilizing changes in wind direction as instrumental variables, it identifies the long-term health impact of average PM2.5 pollutant concentration on China’s elderly population over 1-10 years. The study finds: (1) Air pollution has a significant long-term negative impact on the health level of China’s elderly population. A 10-year average increase of 1μg/m3 in PM2.5 concentration is associated with a 19% increase in the number of chronic diseases, which exhibits a nonlinear trend. (2) Air pollution has a greater long-term impact on women aged 55-65, population of Northern China, and groups with lower levels of education and consumption. (3) Individuals’ avoidance behavior towards air pollution introduces bias in the basic linear regression results, and the negative health impact of long-term exposure to air pollution on the elderly is greater than that of short-term exposure. This paper provides new empirical evidence on the long-term health impacts of air pollution, while offering policy implications for further optimizing response to air pollution and improving the human capital level of the elderly population from an environmental governance perspective.

